# Intermediaries and intermediation in building local transformative capacity for active and sustainable transport

**DOI:** 10.1007/s13280-023-01912-6

**Published:** 2023-09-05

**Authors:** Henna Sundqvist, Anu Tuominen

**Affiliations:** VTT Technical Research Centre of Finland Ltd, Tekniikantie 21, 02044 Espoo, Finland

**Keywords:** Active transport, Intermediaries, Sustainability transformation, Transformative capacity building

## Abstract

Intermediation and transformative capacity building are identified as important issues in sustainability transformations. Yet the connection of these two concepts has not been systematically analysed. This empirical, qualitative case study on active transport in Finland investigates intermediation in building local transformative capacity. The study shows that intermediaries are a heterogeneous group of actors that support transformative capacity building by facilitating the flows of knowledge, linking actors, forming ties across different scales, and supporting visioning and strategic planning. Intermediation manifests in five of the seven elements of local transformative capacity building. Our study, thus, contrasts with previous understanding wherein intermediation is considered only as a criterion for multiform governance. As intermediation is central in building transformative capacity, it should be better acknowledged, particularly by authorities and policymakers to secure legitimacy, operational capabilities, and funding for intermediaries.

## Introduction

The theoretical approaches of Urban and Local Transformative Capacity (e.g. Wolfram 2019; Castán Broto et al. 2019; Tuominen et al. 2022) and Intermediaries in Sustainability Transitions (e.g. Kivimaa et al. 2019; Kanda et al. 2020) have discussed the activities and the role of different actors in sustainability transformations. In both literature strands, the role of intermediaries in supporting transformations is well acknowledged. Intermediaries have been identified as critical in creating momentum for transformation as they link actors and activities, and thus, enhance collaboration, connectivity, and knowledge co-production between different stakeholders, which contributes to building transformative capacity (e.g. Hahn et al. 2006; Kivimaa 2014; Borgström 2019; Wolfram 2019). Despite of recent research, there is a lack of knowledge on how intermediation is organised in practice, particularly in regard with local sustainability transformations.

In the transport context, Tuominen et al. (2022) have developed a framework, comprising seven elements and related criteria, for assessing Local Transformative Capacity (LTC) towards active, sustainable transport. The authors recognised intermediaries positioned between the stakeholders building support as one criterion contributing to multiform governance in the LTC framework for promoting active modes, i.e. walking and cycling. The role and importance of intermediation in the other elements of the LTC, for example related to working and learning across agencies and scales was not, however, discussed and would benefit for further investigation.

In the context of active and sustainable transport, local level actors and activities are crucial in implementing the policy measures and this has recently been highlighted also by the higher-level stakeholders (European Commission 2020). As concerns over negative environmental and social impacts of current urban mobility systems increase, more and more cities are seeking ways to facilitate a transition to more sustainable transport through supporting modes like walking, cycling, and public transport (Loorbach et al. 2021). Even though urban mobility systems in developed countries are nowadays based on a mix of modalities that include public and private options, individual car use is typically the most dominant modality in terms of physical presence, causing problems related to safety issues, parking, congestion, and (air) pollution (Hickman and Banister 2014). The sustainable mobility transition is systemic in nature, requiring changes in vehicles, infrastructures, services, governance, and behaviour of the users, which further increases the challenge.

Local actions in support of active transport, in other words walking and cycling, have been recognised as one of the primary elements supporting the transformation towards sustainable mobility in urban areas (e.g. European Commission 2013, 2020, Headicar 2009; Hiselius and Rosqvist 2016; Panter et al. 2019). Examples of policy instruments that have been implemented in different contexts are walking and cycling strategies and action plans, mobility management programmes and grants, infrastructure development programmes, and local walking and cycling development programmes. All these may include various individual measures, such as new or upgraded cycling and walking lanes, cycle parking, campaigning and education, workplace travel plans, e-bike experiments, etc. Also, the Strategy for a Sustainable and Smart Mobility of the European Commission (European Commission 2020) highlights such activities. Of the individual measures, urban infrastructure has been recognised crucial in enabling active transport (e.g. Félix et al. 2020; Hong et al. 2020), and the role of intermediaries notable in the governance related to infrastructure developments (Moss 2011).

In this paper, our interest lies in the contribution and influences of different actors in sustainability transitions, a topic that is still scarcely studied (Avelino and Wittmayer 2016; Fischer and Newig 2016; Williams et al. 2019). Our research focuses particularly on intermediaries and intermediation supporting active transport. So far, intermediating actors have received only little attention even though their role in communicating, interpreting, and disseminating information and knowledge has been recognised in other contexts, like for example in the energy sector (Kivimaa et al. 2019). Currently, there is still a lack of understanding on for example when interaction between actors becomes intermediation (Kivimaa et al. 2019) and on the potential of intermediation supporting local transformative capacity building. The present empirical study adds to this growing but still limited body of literature by answering to following research questions:

1. What kind of intermediation takes place and between whom in the context of active and sustainable transport?
2. How intermediation supports building local transformative capacity in the field of active and sustainable transport?

## Theoretical framework

### Intermediation and intermediaries in sustainability transformations

Sustainability transformations are fundamental, long-term changes of socio-ecological-technical systems, guided by sustainability goals and policies (Olsson et al. 2014; Markard et al. 2016). Transformations towards sustainability have been conceptualised in several research fields, such as in socio-technical transitions (Markard et al. 2016; Geels 2019) and social-ecological systems (Olsson et al. 2014). Common to these concepts is that the role of intermediation is acknowledged as central to advancing transformations, as intermediaries can enhance and facilitate collaboration, connectivity, trust building, knowledge co-production, and learning (e.g. Hahn et al. 2006; Kivimaa 2014; Borgström 2019; Wolfram 2019).

However, despite such active research, there is not yet a generally agreed definition for intermediation or an intermediary, and these concepts remain contested (Kivimaa et al. 2019; Kanda et al. 2020). According to Kivimaa et al. (2019, p. 1072) intermediaries are ‘actors and platforms that positively influence sustainability transition processes by linking actors and activities, and their related skills and resources, or by connecting transition visions and demands of networks of actors with existing regimes in order to create momentum for socio-technical system change, to create new collaborations within and across niche technologies, ideas and markets, and to disrupt dominant unsustainable socio-technical configuration.’

Many kinds of entities, public or private, governmental or non-governmental act as intermediaries (Hodson and Marvin 2009). These entities entail for example cities, technology transfer offices, industry associations, private companies, clusters, architects and consultants, research and technology organisations and advocacy groups, and there have been several attempts to classify these versatile intermediaries (van Lente et al. 2003; Mignon and Kanda 2018; Kivimaa et al. 2019). Furthermore, different stages of sustainability transformations may require different types of intermediaries (Kivimaa et al. 2019). Common to all these intermediaries is though that they operate within and/or in-between different settings, like geographical and administrative scales, actors or groups of actors, like networks (Kanda et al. 2020).

As pointed out by Kivimaa et al. (2019), there is this still a lack of consensus what intermediation is, how it is defined and when interaction between different entities becomes intermediation. According to van Lente et al. (2003), intermediation is a kind of knowledge brokering, i.e. connecting, translating, and facilitating flows of knowledge between different entities. Several brokerage mechanisms have been identified in the innovation literature, including transfer of resources, matchmaking, and coordination (Spiro et al. 2013). Hodson and Marvin (2009) note that intermediaries connect actors and build networks, and operate on multiple scales (local, national and international). The transition literature also acknowledges a wider range of intermediation activities, including strategic visioning and political influencing (Hodson and Marvin 2009; Kivimaa and Martiskainen 2018; Vihemäki et al. 2020). Kivimaa and Martiskainen (2018) have identified several mechanisms through which intermediaries influence policy development: (1) implementation of pilot projects to demonstrate possibilities and influence political visioning, (2) influencing standard setting and legislation, (3) proving knowledge to support policy development, (4) translating and implementing policy in practice, (5) creating and managing networks to lobby pro-transition policies, and (6) both managing and creating public–private networks that inform the government. Thus, intermediation entails a variety of actions and mechanisms ranging from formal to informal and emergent (Kivimaa et al. 2019).

### Local transformative capacity building and the role of intermediaries

The concept of transformative capacity has its roots in the resilience theory for studying social-ecological systems (SES) (Wolfram et al. 2019). The adaptive capacity, i.e. capacity to strengthen the resilience of a system, was discovered to be insufficient and sometimes even detrimental to SES (Chaffin et al. 2016). In these kinds of cases, more transformative approaches are called for. Transformative capacity aims to ‘create a fundamentally new system when ecological, economic, or social (including political) conditions make the existing system untenable’ (Walker et al. 2004, p. 4). In an urban context, the transformative capacity is defined as ‘the ability of an urban system (inclusive of physical and human dimensions) to reconfigure and move towards a new and more sustainable state’ (Castán Broto et al. 2019, p. 450).

Wolfram (2016) conceptualised transformative capacity and suggested an integrated framework to inform urban policymaking, planning practice, and research. The urban transformative capacity (UTC) framework highlights the role of agency and its different levels (individual, household, organisation, association) and scales (local to global). The framework also emphasises relational aspects, i.e. interactions between actors required for learning and delivering impacts. Building on the seminal work of Wolfram (2016), Wolfram et al. (2019), and Castán Broto et al. (2019), Tuominen et al. (2022) have studied further transformative capacity building in the context of active, sustainable transport and defined the key elements that have built local transformative capacity (LTC) in Finland, Table 1. These key elements are (1) multiform governance, (2) system awareness, (3) future orientation, (4) experimentation, (5) delivering the impacts and implications of the experiments, (6) embedding new solutions and best practices, and (7) working and learning across agencies and scales.

**Table 1:** The framework for assessing local transformative capacity (LTC) towards active, sustainable transport (Tuominen et al. 2022)

Element of LTC	Criteria
Multiform governance (LTC 1)	Transformative leadership driving the change Commitment of local government to promote active modes Collaboration of various stakeholders in building support to active modes Intermediaries and hybrid actors positioned between the stakeholders to build support Participation of the public in planning or decision-making processes, recognising and addressing different social needs
System awareness (LTC 2)	Understanding and monitoring of the state of the transport system Recognition of path dependencies, systemic barriers that need to be overcome
Future orientation (LTC 3)	Building collective vision for change, strategy or future pathways
Experimentation (LTC 4)	Implementing new solutions or ideas that seek to challenge the established policies, technologies or social practices
Delivering the impacts and implications of the experiments (LTC 5)	Assessment of the impacts, implications and social learning delivered by the experiments Availability of methods, criteria and processes for assessing the impacts
Embedding new solutions, best practices (LTC 6)	Sharing lessons learned, knowledge, expertise and offering direct advice or support Replicating good practices in different settings, embedding changes in institutional settings New regulation, guidance or recommendations established as a result of a good experiment
Working and learning across agencies and scales (LTC 7)	Activities of collaboration and capacity development at local level: involving various and multiple stakeholders in knowledge production processes, social learning Activities of collaboration and capacity development across geographical or political-administrative levels, social learning

Transformative capacity building relies on fostering creativity and supporting connectivity through multi-actor collaboration, goal setting, trust building, and knowledge co-production and sharing, in all of which intermediation plays an important role (Wolfram et al. 2019). In the case of local sustainability initiatives, the role of intermediaries is vital to support collaboration and knowledge transfer between separate initiatives and the actors needed for transformative capacity building (Borgström 2019). If the roles and requirements of intermediaries are not sufficiently acknowledged or understood, the connectivity between actors may remain poor, constraining the collective re-orientation and re-alignment of a system (Wolfram et al. 2019). Overcoming this gap requires supporting existing intermediaries and developing new ones through developing new governance models and securing the appropriate funding (Borgström 2019; Wolfram et al. 2019).

Our contribution strengthens the understanding on what kind of intermediation takes place and is required, particularly for building local transformative capacity in the context of active and sustainable transport.

## Materials and methods

### Case description—active transport in Finland

The Ministry of Transport and Communications of Finland put together and published Finland's first Walking and Cycling Promotion Programme in 2018. The aim of the programme is to improve the circumstances for walking and cycling in Finnish municipalities, to support the reduction of greenhouse gas emissions from transport, and further promote public health in Finland by presenting ten key areas for action. The programme complements the first National Strategy for Walking and Cycling (2011) and Action Plan (2012). The ambitious target of the programme is a 30% increase in the number of walking and cycling trips by 2030 (compared to 2018 level). The 30% increase means 450 million additional walking and cycling trips in 2030, and a 35–38% modal share for walking and cycling instead of the current 30%. The programme presents the frame and basis for municipalities and other national actors to build their future activities on.

Cooperation between different sectors is essential in promoting walking and cycling. Besides the state administration, municipalities are key actors in Finland in promoting and implementing walking and cycling measures and carrying out these activities in collaboration with local stakeholders and citizens. Since the promotion of active modes touches different organisational sectors of municipalities, such as transport and urban planning, construction, traffic management, recreation and sports, public health, and education, collaboration across sectors is decisive in realising mutual benefits.

During the past decade, municipalities and regional authorities have been the main responsible actors in financing and implementing walking and cycling measures. Besides municipalities and regional authorities, in 2010–2019 the government financed over 100 local walking and cycling projects through a national mobility management programme, government grants, and a walking and cycling programme. Despite their modest financial contribution, the projects have been important initiators especially for local cycling promotion and actor collaboration. Four types of projects have been funded: (1) campaigns, marketing, communication, and events; (2) local strategies and action plans; (3) pilot projects in organisations (e.g. on using e-bikes for commuting); and (4) new service development (Liikennevirasto 2018). The topics put forward a key mobility management message that significant change in mobility choices cannot be achieved simply by building new infrastructure for active modes. There is also a need for influencing attitudes and behaviour and raising the quality level of existing networks, urban form, and service networks that promote walking and cycling.

In our analysis, we focused on intermediation and intermediaries at national level and in six municipalities that have shown interest and progress in developing conditions for active modes during the past decade. The municipalities include the capital city, Helsinki, the third largest city in Finland, Tampere, Turku on the south-west coast, Oulu in the northern part of the country, Jyväskylä in central Finland, and Joensuu in the east.

### Data collection

The data and analysis frame of this exploratory study builds partly on our previous qualitative study (cf., Tuominen et al. 2022), in which we also employed a purposive, iterative sampling, and data collection approach (cf., Drisko and Maschi 2015). In this paper, the data include interviews and a corpus of documents, including web pages, and other written documents in electronic format. The data were collected during the period 2020–2022. The primary data consist of (1) 10 interviews of persons with expertise on active and sustainable transport promotion, (2) web pages of organisations involved in active and sustainable transport promotion, and (3) discussions with representatives of two civil society organisations promoting active transport. The secondary data consist of reports on walking and cycling projects funded by the national mobility management (MM) programme, governmental MM grants or governmental walking, and cycling programmes during 2010–2021, and local walking and cycling action plans.

We applied a semi-structured interview protocol (cf., Yin 2014) covering the following main themes: (1) Successful cycling and walking initiatives in the municipality during the past decade and the reasons for success; (2) Obstacles to implementing the planned actions; (3) Processes and practices within the municipality and with stakeholders in promoting active transport; (4) The role of the three governmental programmes in promoting active modes in the municipality during the past decade; and (5) The future of active transport in Finland and in the region: opportunities and threats. The interviewees, listed in Table 2, were identified by visiting the web pages of organisations focusing on walking and cycling promotion and reading the reports on walking and cycling projects (i.e. the secondary data). Previous contacts with experts active in mobility management in Finland were also used.

**Table 2:** Interviewed organisations

Organisation	Number of interviews
Municipality, transport planning, and construction	2
Municipality, sustainable mobility activities	3
Regional expert organisation promoting sustainable development	1
Local civil society organisation promoting sustainable development	1
Public Transport Authority	1
Consulting company specialised in sustainable mobility services	1 (2 representatives)
Civil society organisation promoting cycling	1

While the interview guide covered a wider range of topics on the promotion of active transport than mere intermediation activities, it provided us rich data on different actors and their activities and allowed us to identify entities that intermediate even though this may not be their primary activity. The data, thus, also allowed us to study mechanisms of intermediation. The interviews were conducted in Finnish in 2020 and 2021, recorded, and transcribed. Two interviewees were present in nine interviews. One of the interviews had only one interviewer. The length of the interviews ranged from 60 to 90 min. As the interviews were conducted in Finnish, the quotations given in the results have been translated into English by the authors.

### Coding and qualitative analysis

Our analysis is based on a directed approach of qualitative content analysis, where previous research and theory guided our initial thematic coding and analysis (Hsieh and Shannon 2005). The data analysis focused on the primary data (i.e. interview transcripts, notes, and documentary sources). In the first stage, we identified activities and mechanisms of intermediation and based on those, we identified the key intermediaries in the context of sustainable and active transport.

The coding and analysis of intermediation mechanisms began with using the mechanisms described in the previous literature as a frame of reference (e.g. Hodson and Marvin 2009; Kivimaa and Martiskainen 2018; Kivimaa et al 2019; Vihemäki et al. 2020) (see “Theoretical framework” section). The final coding categories were iteratively formed, based on our data, including mechanisms of knowledge transfer, matchmaking, coordination, and policy influencing. During the third round of coding, we focused on identifying how intermediation supports the local transformative capacity building. In this part, we used the frame developed in our previous study (cf. Tuominen et al. 2022, p. 5) to systematically code and analyse how intermediation is present in the seven elements of transformative capacity building in the context of sustainable transport: (1) multiform governance, (2) system awareness, (3) future orientation, (4) experimentation, (5) delivering the impacts and implications of the experiments, (6) embedding new solutions, best practices, and (7) working and learning across agencies and scales. We used a set of 16 criteria of LTC (see Table 1) to guide our analysis in identifying the relevance of intermediation activities to a specific element of LTC.

The coding was done simultaneously by both authors, and the categories and findings were agreed upon during the process. Throughout the coding stage, the coded data were organised using an Excel spreadsheet.

## Results

### Intermediation mechanisms contributing to local transformative capacity building

We recognised four mechanisms of intermediation as being central to the active and sustainable transport context. These are: (1) Knowledge transfer, (2) Matchmaking, (3) Coordination, and (4) Policy influencing. The first of these connects, translates, and facilitates flows of knowledge and data between different parties. We call this mechanism *knowledge transfer*. Based on our data, there are several active intermediaries transferring data and knowledge in Finland. These are civil society organisations such as the Cyclists’ Federation, national and regional expert organisations, local associations, and sometimes also consultants promoting sustainable development. The knowledge transfer typically occurs between members of a network of, e.g. organisations and municipalities promoting active and sustainable transport, and between collaborating partners or municipalities and citizens within a project or experiment. The means of knowledge transfer are seminars, an annual national cycling conference (VeloFinland), the annual European Mobility Week, webinars, newsletters, regular meetings with municipalities, informing about potential funding opportunities for active transport (e.g. annual Mobility Management state grant call for municipalities), and participation in development projects and experiments.

Knowledge transfer builds local transformative capacity in two ways. First, intermediaries contribute to multiform governance as they translate and facilitate flows of knowledge (e.g. recent scientific knowledge and best practices) among different municipal actors and organise campaigning and training with various citizen groups such as youth, the elderly, children, or families, for example through cycling with small children. Local training activities are considered very important for sustainable and active mobility transitions, as can be seen from the following comment by a representative of a local sustainable development association: ‘Changes cannot be made by telling people that this is how we do it. People need to do and experience it by themselves, and that we see as a must’.

Second, knowledge transfer is vital in experimentation, which builds local transformative capacity, as intermediaries collaborate with several stakeholders in experiments and share information as well as responsibilities. The national Mobility Management state grant is here a key means of project funding through which experimentation takes place. Particularly civil society organisations, like local cycling associations and local sustainable development associations, work actively between municipalities and citizens by participating or arranging experiments, where citizens can, for example, learn on and test active modes (e.g. electric bikes). Besides the abovementioned organisations, civil servants also play a key role in local experiments through information sharing, increasing media visibility, and campaigning in favour of sustainable, active transport.

The second mechanism of intermediation facilitates the formation of direct ties between one party and another, that is, *matchmaking*. In this mechanism, national level civil society organisations are key intermediaries and matchmaking can also form a core function of their activities: ‘We create cooperation opportunities, convey information, and offer practical solutions to build a sustainable society’.

In addition, civil servants seem to have a significant role in connecting actors and building collaboration between municipality departments (e.g. transport planning, construction, recreation, and health promotion) but also between municipalities to implement actions that promote active modes. The importance of such matchmaking within a municipality has been highlighted by a civil servant as follows*: *‘The bigger the organisation, the more different stakeholders there are, which should be brought together to work together for a common goal’*.* Consequently, matchmaking also seems to build local transformative capacity by actively supporting multiform transport governance.

The third mechanism facilitates parties to interact without forming direct ties, by performing *coordination.* We found that in the context of active mode promotion, intermediation takes place most often between members of a network. Civil society organisations represent one type of network coordinator. The cyclists’ federation coordinates the local associations, the Network of Cycling Municipalities Association manages the activities between municipalities or between departments within a municipality. The national sustainable development expert organisation shows even wider coordination by connecting all kinds of actors interested in promoting active modes within a smart mobility network (i.e. VILI network) that it manages. The purpose of the network is to increase cooperation and information exchange. This national expert organisation also coordinates the activities of the annual Mobility Week and manages the annual Mobility Management government grant process. Similarly, some regional expert organisations coordinate regional sustainable mobility activities. Further, as our interviews revealed, a regional public transport agency can also take an intermediary role, for example in coordinating the development of cycle parking and Park/Cycle-and-ride services or coordinating the creation of marketing strategy and cycling brand between the region’s municipalities.

'We have tried to promote, in particular, the bike-and ride facilities connected to metro and commuter train transport in cooperation with the municipalities. [Our company] is not the entity that builds the infrastructure, it is always the municipality that is responsible for the park- and bike-and ride facilities in its own area. We coordinate the activities in the Helsinki region, partly through the regional transport planning work.'

The above examples show that coordination builds transformative capacity particularly through working and learning across agencies and scales.

Finally, the fourth mechanism connects transition visions and demands of networks of actors with existing regimes to create momentum for system change. This we consider as *policy influencing*. The civil society organisations, especially the Cycling Federation, local cycling associations, and Network of Cycling Municipalities association have been active policy influencers, the former fulfilling its role as an interest group: ‘The Cycling Federation represents cyclists in national decision-making, has regular meetings with the decision-makers, and participates in procedures for the drafting of legislation’.

Intermediation may simultaneously take place through several mechanisms. For example, knowledge transfer and policy influencing may take place as intermediating actors support and influence vision building, and in turn contribute to capacity building for multiform transport governance and future orientation. ‘We provide building blocks to municipalities for creating their mobility visions’, as pointed out an interviewee from a civil society organisation. Policy influencing also takes place between citizens and municipality civil servants and the council and between municipalities and national transport agencies and ministries and is closely linked with the mechanism of knowledge transfer. Regular meetings with decision makers and participation in processes for drafting legislation are examples of means for policy influencing. The mechanisms of intermediation with linkages to the key elements of local transformative capacity building (see also “Local transformative capacity building and the role of intermediaries” section) are summarised in Table 3.

**Table 3:** Mechanisms, actors, and intermediation activities in relation to the elements of local transformative capacity building (in brackets) in active and sustainable transport

Mechanism of intermediation	Groups of actors intermediating	Intermediation activities	Present in a specific element of local transformative capacity building
Knowledge transfer	Civil society organisations National and regional expert organisations Consultants	Arranging and participating to networking events, like annual Velo Finland conference, seminars, and webinars, disseminating information through newsletters, participating to development projects and regular meetings	Multiform governance (LTC 1) Experimentation (LTC 4) Delivering impacts of experiments (LTC 5) Working and learning across agencies and scales (LTC 7)
Matchmaking	Civil society organisations Civil servants	Connecting actors to facilitate intra-organisational discourse	Multiform governance (LTC 1) Working and learning across agencies and scales (LTC 7)
Coordination	Civil society organisations National and regional expert organisations Public transport agencies Civil servants	Coordinating VILI network Mobility week national coordination Developing of regional cycle and ride services Creation of a regional cycling brand	Working and learning across agencies and scales (LTC 7)
Policy influencing	Civil society organisations	Having regular meetings with decision makers Participation in processes for drafting legislation and policies	Multiform governance (LTC 1) Future orientation (LTC 3) Working and learning across agencies and scales (LTC 7)

### Intermediaries and intermediation between actors and scales

In our analysis on intermediation activities and mechanisms, we were able to identify five groups of actors that carry out aforementioned intermediation activities. It is important to emphasise that while not all the identified actors are intermediaries as their primary function, they still intermediate through several mechanisms (*see also *Table 2). The key intermediating actors include civil servants in municipalities; regional and national expert organisations promoting sustainable development; civil society organisations, such as a national cyclists' federation, local cycling associations, a local sustainable development association, and an association for networking municipalities towards active transport; and public transport agencies and consultants specialised in sustainable mobility services and sustainable development, see Table 4.

**Table 4:** Groups of intermediaries and examples of organisations in active and sustainable transport in Finland

Intermediary	Examples of organisations	Aims and activities on active and sustainable transport
Civil servants	Municipality of Oulu Municipality of Turku	To promote smooth implementation of cycling and walking infrastructure and activities
Regional and national expert organisations for sustainable development	Motiva	To support the public sector, businesses, municipalities, and consumers with information, solutions, and services that allow them to make resource-efficient, effective, and sustainable choices
	VALONIA	To support regional municipalities, companies, and citizens in promoting sustainable development, including mobility management activities
Civil society organisations	Jyväskylä Sustainable Development Association	To promote and facilitate sustainable choices in the daily lives of citizens
	Finnish Cyclists’ Federation	To increase the modal share of cycling in Finland
	Network of Cycling Municipalities Association	To promote cycling, cycling culture, and cycling conditions in Finland, to increase the interaction between municipalities, state administration, companies, and various organisations in cycling and walking promotion, and to disseminate information to support development work
	Jyväskylä Cyclists Association	To promote cycling and better cycling conditions in Central Finland
Public transport agencies	HRT Helsinki Region Transport	To organise public transport and offer sustainable mobility choices for citizens and companies
Consultants	Valpastin	To promote sustainable transport through development plans and projects with municipalities and companies and in collaboration with service providers

Intermediation contributes to local transformative capacity building particularly by connecting actors and entities at different scales*.* Intermediaries are active not only at local, regional, or national level, but can work across these different scales, share knowledge, and connect actors. The importance of this cross-scale interaction is also highlighted by the intermediaries: ‘I think it cannot be overemphasised that you can't just stay in your own sector, but truly do it [transformation] together’.

Building on the work of Kanda et al. (2020) on intermediation between different settings, we identified the following four settings for intermediation in the context of active and sustainable transport transition: (1) between departments or actors within an entity, (2) between individual entities, (3) between entities in a network, and (4) between individual entities and a network, Table 5. *Intermediation between departments or actors within an individual entity* takes place, for example, within a municipality between different departments (e.g. transport planning, construction, recreation, and health promotion) or between a council and a municipality department, and it crosses mainly administrative scales.

**Table 5:** Intermediation between different entities and scales in sustainable and active transport. Visualisation elaborated from Kanda et al. (2020)

Setting	Intermediation between departments or actors within an entity	Intermediation between individual entities	Intermediation between entities within a network	Intermediation between individual entities and a network
Visualisation				
Intermediaries	Civil servant, civil society organisation, consultants	Consultant, civil servant, civil society organisation, regional expert association	Civil society organisation, national and regional expert organisation, public transport agency	Civil society organisation, public transport agency
Entities	Municipality department, Municipality council, Regional centre of development	Project partner, National authority, Municipality council	Municipalities, Organisations in a national network (VILI, cycling municipalities)	Citizens, Municipality, National authority, Research organisation, Network of cycling municipalities
Examples of intermediaries (I), entities (E), sub-units of an entity (X, Y), and networks (N)	Municipality department (X)—Civil servant (I)—Municipality department (Y) Council (X)—Civil society organisation (I)—Municipality department (Y)	Project partner (E)—consultant/civil servant (I)—project partner (E) Project partner (E)—consultant (I)—local/national authority (E) Municipality council (E)—Civil servant (I)—Centre for economic development, transport and the environment (E)	Expert organisation (I)—organisations (E) in a national network (N) (e.g. VILI network) Civil society organisation (I)—municipalities (E) in a national network (N) Regional expert organisation (I)—municipalities (E) in a regional network (N)	Municipality (E)—Cycling association (I)—Cyclists in a municipality (N) Municipality/ National authority (E)—Local SD association (I)—Citizens in a municipality (N) Research organisations (E)—Civil society organisation (I)—Cycling municipalities (N)
Crossing scales	Local	Local–regional–national	National–regional–local	National–local, local

*Intermediation between individual entities* can take place within or across different organisational, sectoral, and geographical scales. For example, in projects funded by the national Mobility Management Grant, civil servants or consultants acting as intermediaries may mediate between the project partners from different organisations. Intermediation may also cross local and regional scales, as in the case when a civil servant intermediates between a municipality council and a regional centre of economic development, transport, and environment (i.e. ELY centre).

*Intermediation between entities within a network* can cross all geographic scales (local, regional, national). In this case, intermediation in supporting active and sustainable transport takes place, for example, between organisations in a national network, as in the case of the VILI network which covers the whole of Finland, or it can be between municipalities in a regional network (i.e. regional councils, Helsinki region) or between municipalities across Finland in a network of cycling municipalities.

The fourth type of setting where intermediation is observed is *between individual entities and a network*. In this setting, intermediation can cross several geographic scales, like local and national, or take place within a certain scale, like in cities. The work of cycling associations sharing information between a network of cyclists in a municipality and the municipality illustrates local level intermediation. Another example of intermediation between individual entities and a network is knowledge transfer between research organisations and the network of cycling municipalities mediated by a civil society organisation. The aim of intermediation is to share recent research findings on active and sustainable transport with municipalities that want to promote cycling and walking.

## Discussion

Intermediation is acknowledged as vital in building transformative capacity and fostering sustainability transformations. Our aim was to shed light on how in practice and through which mechanisms intermediation contributes to local transformative capacity building in the context of active and sustainable transport. Based on our analysis we propose that intermediation for building transformative capacity is brokering, that manifests through mechanisms of knowledge transfer, coordination, matchmaking, and policy influencing, and facilitates the flows of knowledge, links actors, forms ties across different scales, and supports visioning and strategic planning with an aim to increase the ability of a local system to reconfigure and move towards a more sustainable state.

Our study showed that five groups of actors, from individual civil servants and consultants to civil society organisations and national expert organisations, perform a variety of activities that can be identified as intermediation, which supports transformative capacity building. What seems to be common to this heterogeneous mix of entities is that all intrinsically aim to promote active and sustainable transport by creating favourable conditions for the growth of cycling and walking in Finland. Furthermore, these entities are all highly networked, sometimes even with other intermediaries. Another interesting feature is that intermediary organisations or individual actors mainly act within the existing system and are, therefore, according to Kivimaa et al. (2019), regime intermediaries. The promotion of walking and cycling intertwines with some of the activities and targets of the national social and health care regime, yet our study did not indicate intermediation between these two regimes, nor intermediaries active beyond the transport regime. Furthermore, instead of radical or disruptive transformations, intermediation activities seem to support gradual change.

Most entities that we identified as intermediating do not necessarily perceive themselves primarily as intermediaries. Of the five groups identified, national expert organisations promoting sustainable development and civil society organisations have been most active in performing intermediation activities. This is an expected result since both actors are intermediaries as their primary function. Instead, a novel finding is that local civil servants perform intermediation through several mechanisms, in particular matchmaking and coordination, and across municipal sectors which is important for advancing transformations within municipalities.

In line with previous literature (cf. Spiro et al. 2013; Kivimaa and Martiskainen 2018), we identified four mechanisms of intermediation: knowledge transfer, matchmaking, coordination, and policy influencing. Of these, knowledge transfer and policy influencing have been the most often applied mechanisms in the context of local transformative capacity building for active and sustainable transport in Finland. It is fair to ask how these mechanisms and activities of intermediation differ from mere policy making or implementation. As pointed out by Moss (2011), intermediation may have political nature and intermediaries have their own agendas. Our findings support this view and show that intermediation is present in multiform governance and the implementation and development of policies that support walking and cycling. To distinguish mere interaction or lobbying from intermediation, we found that intermediation, besides often being goal oriented, aims at facilitating bi- or multidirectional flows of knowledge, in line with van Lente et al. (2003). Furthermore, in many cases the desired outcome of intermediation was learning and consequent practical action or behavioural change supporting sustainable transport transition.

As the previous literature shows, intermediation is already acknowledged as an important factor in transformative capacity building. Our study exemplifies how intermediaries and intermediation contribute particularly to local transformative capacity building in practice. Intermediation is central to connecting a variety of actors across different scales (LTC element 7), and the identified intermediaries operate within a scale and/or between multiple scales (local, regional, national), across organisational structures and through all the identified four mechanisms. Furthermore, our findings revealed (see Table 3) that intermediation is relevant and can contribute to the building of most of the elements of LTC. This contrasts with previous understanding such as the framework of urban transformative capacity (Wolfram 2016) and the modified framework for local transformative capacity (LTC) in the transport context (Tuominen et al. 2022), wherein intermediation is considered only as a criterion for multiform governance.

Furthermore, the results indicate that intermediaries contribute to building future-oriented capacities (LTC element 3), particularly in municipalities, and to supporting a multiform transport governance in general (LTC element 1). Here, intermediation mechanisms are comprised of knowledge transfer in different events, matchmaking, and especially policy influencing via participation in various discourse and policy and regulatory processes. Intermediation in experimentation (LTC element 4) and delivering the impacts of experiments (LTC element 5) through knowledge transfer, for example by sharing information as well as responsibilities in local project contexts, are also present in building local transformative capacity for active and sustainable transport.

Despite the active role of intermediation and intermediaries in experimentation supporting and promoting cycling and walking in municipalities, we recognised that intermediation is largely missing in embedding new solutions and best practices (LTC element 6), even though it could have a major impact on the scale-up and integration of new solutions and practices with the present activities and processes promoting active modes. Our analysis identified that intermediating coordination to support the evaluation processes of governmental programmes and grants supporting active modes should be strengthened to initiate wider deployment and embedding of the results. As pointed out by Borgström (2019), intermediation is important in ensuring that the learning and best practices are not lost but transferred to local and national level authorities, thus, influencing policies and supporting transformation.

Based on our research on local sustainability activities and initiatives supporting walking and cycling, we argue that intermediaries connect a versatile set of entities and by doing so enhance collaboration and knowledge sharing across agencies and geographical scales, as also suggested by Hodson and Marvin (2009). Especially actors who carry out intermediation as their primary function have managed to create, over the past ten years, permanent networks of entities that are interested in and actively working towards the promotion of active modes. However, our empirics also indicate that most organisations that intermediate are not intermediaries as their primary function, and their role as intermediaries is not self-evident. Moss (2011) argues that the function of intermediation is more important than defining whether an actor is an intermediary or not. However, based on our results, we emphasise that both intermediation activities and entities intermediating should be better acknowledged, particularly by the authorities and policy makers. This recognition would be important to secure legitimacy, operational capabilities, and funding for intermediaries, as also highlighted by Borgström (2019) and Wolfram et al. (2019).

To conclude, this study sheds light on intermediation and intermediaries in building local transformative capacity for active and sustainable transport. We found that intermediaries are actors and organisations, such as civil servants, consultants, and civil society organisations, that support cycling and walking, which are essential for advancing sustainable mobility transformation. The study shows that intermediation takes place and is relevant to local transformative capacity building through four key mechanisms of intermediation.
